# Malaria in French Guiana Linked to Illegal Gold Mining 

**DOI:** 10.3201/eid2202.151292

**Published:** 2016-02

**Authors:** Vincent Pommier de Santi, Aissata Dia, Antoine Adde, Georges Hyvert, Julien Galant, Michel Mazevet, Christophe Nguyen, Samuel B. Vezenegho, Isabelle Dusfour, Romain Girod, Sébastien Briolant

**Affiliations:** Army Center of Epidemiology and Public Health, Marseille, France (V. Pommier de Santi, A. Dia);; Direction Interarmées du Service de Santé en Guyane, Cayenne, French Guiana (V. Pommier de Santi, G. Hyvert, J. Galant, M. Mazevet, C. Nguyen, S. Briolant);; Institut Pasteur, Cayenne (A. Adde, C. Nguyen, S.B. Vezenegho, I. Dusfour, R. Girod, S. Briolant);; Institut de Recherche Biomédicale des Armées, Brétigny-sur-Orge, France (C. Nguyen, S. Briolant)

**Keywords:** malaria, French Guiana, illegal gold mining, parasites, anopheles mosquitoes, Anopheles spp., vector, plasmodium falciparum, plasmodium vivax

**To the Editor:** French Guiana, an overseas territory of France and part of the European Union, is located on the northeast coast of South America (Figure). During 2008– 2014, the number of malaria cases reported in French Guiana drastically decreased (*1*). The littoral area (≈30 km–wide Atlantic Ocean coastal band between the cities of Awala-Yalimapo and Ouanary) and the lower part of the Maroni River bordering Suriname (between the cities of Maripasoula and Saint-Laurent du Maroni) are considered malaria free, but this status may not reflect malaria transmission in the inland rainforest (*2*–*4*). Since 2008, French Armed Forces have been involved in military operations to control and reduce illegal gold mining activities in forested areas. Soldiers and military policemen usually spend 1–3 weeks in illegal gold mining sites in remote rainforest areas before returning to the littoral area or to bases on rivers bordering Suriname and Brazil. Despite malaria prevention strategies (*5*), these deployments have resulted in several outbreaks and increased malaria incidence among French forces (*6*). Most malaria episodes occurred during or just after deployments, so presumed locations of exposure can be easily identified.

**Figure:**
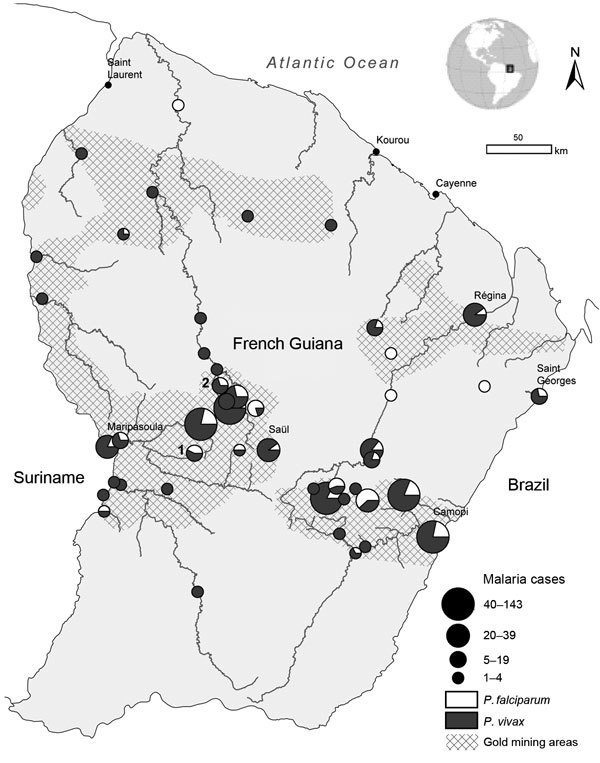
Geographic distribution of presumed places of exposure for 742 single-infection *Plasmodium vivax* (586) and *P. falciparum* (156) malaria cases reported among French Armed Forces in French Guiana, 2008–2014. Numbers on map show illegal gold mining sites where entomologic investigations were conducted; 1 indicates Eau Claire; 2 indicates Dagobert.

Information about malaria cases was collected during 2008–2014 by the French Armed Forces’ epidemiologic surveillance system by using a mandatory, specific form that captured putative place of malaria exposure and biologic data for case-patients (*6*). Geographic coordinates of presumed places of contamination were uploaded into a geographic information system (ArcGIS; http://www.esri.com/software/arcgis/) to produce a malaria distribution map.

During 2008–2014, a total of 1,070 malaria cases were reported to the French Armed Forces’ epidemiologic surveillance system. *Plasmodium vivax* accounted for 78.8% (843/1,070), *P. falciparum* for 18.0% (193/1,070), and mixed infection (*P. vivax* and *P. falciparum*) for 3.2% (34/1,070). Places where malaria exposure occurred were identified for 742 cases of single malaria (586 *P. vivax* and 156 *P. falciparum*) infections (Figure). Cases occurring along the Maroni and Oyapock Rivers delimiting the frontiers with Suriname and Brazil, respectively, accounted for 25.3% (188/742). The other cases (74.7%, 554/742) were associated with exposures during military operations in illegal gold mining sites.

Entomologic investigations were conducted in 2 malaria epidemic locations where French forces were deployed: Eau-Claire and Dagobert. Collected *Anopheles* spp. mosquito specimens were identified by using morphologic keys specific to the Guyana Shield, a geomorphologic formation underlying French Guiana and other areas (*7*). Nonidentifiable *Anopheles* mosquito specimens were further identified molecularly (*8*). PCR products from the internal transcribed spacer 2 gene were sequenced, and *Anopheles* species were identified by comparing sequences to those in GenBank (http://www.ncbi.nlm.nih.gov/genbank/) by searching with BLAST (http://blast.ncbi.nlm.nih.gov/Blast.cgi). Testing for *P. falciparum* and *P. vivax* infections was conducted for all *Anopheles* spp. specimens by using nested PCR, as described (*9*). 

In May 2013, a malaria outbreak occurred 1 month after military deployment of 100 soldiers at Eau Claire (3.56075°N, −53.21268°E; Figure), where 1 Mosquito Magnet trap (Woodstream Corporation, Lititz, PA, USA) baited with octenol was used to sample *Anopheles* mosquitoes during April 22–May 12, 2013 (*10*). The attack rate among the soldiers was 5.0% (5/100): 4 *P. vivax* and 1 *P. falciparum* malaria cases. Fifty-three *Anopheles* mosquito specimens were caught during the 20 days before the outbreak and identified as comprising 4 species (Technical Appendix Table). *P. falciparum* infection was detected in 2 *Anopheles* species: 1 (12.5%) of 8 *An. ininii* and 1 (5.0%) of 19 *An. nuneztovari s.l.* mosquitoes collected; *P. vivax* infection was found in 1 (5.5%) of 19 *An. nuneztovari s.l.* mosquitoes.

In September 2013, another malaria outbreak occurred 3 weeks after the deployment of 15 soldiers in Dagobert (4.06028°N, −53.70667°E; Figure). The attack rate among these soldiers was 53.3% (8/15): 7 *P. vivax* infections and 1 co-infection with *P. vivax* and *P. falciparum*. Mosquitoes were collected 3 months later by using human landing catches during 5 consecutive days. The area had been free of illegal gold mining activities since the 15 soldiers were deployed. A total of 321 *Anopheles* mosquitoes were collected in this location; 95.6% were identified as the same 4 species as in the Eau Claire mosquito collection (Technical Appendix Table). Only 1 specimen (0.4%, 1/282), *An. darlingi* mosquito, was infected with *P. vivax*.

These results suggest a high level of malaria transmission involving *An. darlingi* and other *Anopheles* species as primary vectors of malaria in the rainforest. The findings probably highlight malaria hyperendemicity in communities of undocumented gold miners, who are often mobile and pose a challenge for controlling malaria and other infectious diseases in the region. Indeed, these gold miners could reintroduce malaria in areas where competent vectors exist in the coastal part of French Guiana and in Surinam and Brazil, which border French Guiana. This potential for transmission could seriously threaten the success of malaria elimination programs in the Guiana Shield. Further studies are needed to better evaluate malaria epidemiology in these undocumented populations to determine how best to adapt strategies to control malaria transmission in this subregion of South America.

Technical Appendix The distribution of mosquitoes sampled by sampling sites and *Plasmodium *infection rates of the 374 *Anopheles *mosquitoes caught in the French Guiana forest, 2013
