# Supplementary material for: Malaria in French Guiana Linked to Illegal Gold Mining
Source: Emerg Infect Dis. 2016 Feb;22(2):344–6. doi: 10.3201/eid2202.151292 (PMC4734546; doi:10.3201/eid2202.151292)
Supplement: Technical Appendix — The distribution of mosquitoes sampled by sampling sites and Plasmodium infection rates of the 374 Anopheles mosquitoes caught in the French Guiana forest, 2013 [file 15-1292-Techapp-s1.pdf]

Article DOI: http://dx.doi.org/10.3201/eid2202.151292

Malaria in French Guiana Linked to Illegal Gold Mining

Technical Appendix

Technical Appendix Table. Distribution of mosquitoes sampled by sampling sites and *Plasmodium* infection rates of the 374 *Anopheles* mosquitoes caught in the French Guiana forest, 2013

	Dagobert*			Eau Claire†		
	Collected mosquitoes, no. (%)	*Plasmodium* species, no. infected (infection rate, %)		Collected mosquitoes, no. (%)	*Plasmodium* species, no. infected (infection rate, %)	
Anopheles species		*P. vivax*	*P. falciparum*		*P. vivax*	*P. falciparum*
An. darlingi	282 (87.8)	1 (0.4)	–	2 (4.0)	0	0
An. nuneztovari s.l.	17 (5.3)	0	–	19 (36.0)	1 (5.0%)	1 (5.0)
An. triannulatus s.l.	7 (2.2)	0	–	24 (45.0)	0	0
A. ininii	1 (0.3)	0	–	8 (15.0)	0	1 (12.5)
An. spp.‡	14 (4.4)	0	–	0	0	0
Total	321 (100.0)	1 (0.3)	–	53 (100.0)	1 (2.0)	2 (4.0)

*Human-baited landing 3 months after a malaria outbreak among French military personnel.
†*Anopheles* mosquitoes sampling 1 month before a malaria outbreak among French military personnel.
‡Species unidentified by morphologic or molecular tests.
